# Clinical Applications of Anterior Segment Optical Coherence Tomography in Managing Phakic and Secondary Intraocular Lens Implants: A Comprehensive Review

**DOI:** 10.3390/diagnostics15182385

**Published:** 2025-09-19

**Authors:** José Ignacio Fernández-Vigo, Bárbara Burgos-Blasco, Lucía De-Pablo-Gómez-de-Liaño, Ignacio Almorín-Fernández-Vigo, Pedro Arriola-Villalobos, Diego Ruiz-Casas, Ana Macarro-Merino, José Ángel Fernández-Vigo

**Affiliations:** 1 Department of Ophthalmology, Hospital Clínico San Carlos, San Carlos Health Research Institute (IdISSC), 28040 Madrid, Spain; bburgos171@hotmail.com (B.B.-B.); pedro_arriola@hotmail.com (P.A.-V.); 2 Department of Immunology, Ophthalmology and ENT, Faculty of Medicine, Complutense University of Madrid, 28040 Madrid, Spain; 3 Centro Internacional de Oftalmología Avanzada, 28010 Madrid, Spain; ana.macarro@fernandez-vigo.com (A.M.-M.); jose.fernandez-vigo@fernandez-vigo.com (J.Á.F.-V.); 4 Department of Ophthalmology, Hospital Universitario 12 de Octubre, 28041 Madrid, Spain; depablo.lucia@gmail.com; 5 Department of Immunology, Ophthalmology and ENT, Faculty of Optics, Complutense University of Madrid, 28037 Madrid, Spain; 6 Centro Internacional de Oftalmología Avanzada, 06011 Badajoz, Spain; ignacioalmorin@gmail.com; 7 Department of Ophthalmology, Hospital Ramón y Cajal, 28034 Madrid, Spain; druizcasas@hotmail.com; 8 Department of Medical-Surgical Therapy, Faculty of Medicine, University of Extremadura, 06006 Badajoz, Spain

**Keywords:** optical coherence tomography, intraocular lens, phakic lenses, secondary intraocular lens implant, aphakic, refractive surgery, cataract

## Abstract

Anterior segment optical coherence tomography (AS-OCT) has emerged as a crucial imaging technique in ophthalmology, particularly for evaluating intraocular structures and the behavior of phakic and secondary intraocular lenses (IOLs). This narrative review summarizes the latest findings and clinical applications of OCT regarding phakic and secondary IOLs, focusing on their effectiveness, safety, and factors influencing performance. Through a comprehensive analysis of current literature, we explore how OCT facilitates the assessment of IOLs on key anatomical parameters—such as vault, angle configuration, lens centration, tilt, and haptic positioning—essential for optimizing surgical outcomes and minimizing postoperative complications. In phakic IOLs, including posterior chamber lenses such as the Implantable Collamer Lens (ICL, STAAR Surgical, Monrovia, CA, USA) and iris-fixated lenses, such as Artiflex (Ophtec BV, Groningen, The Netherlands), OCT enables precise evaluation of the anterior segment, aiding both candidate selection and long-term monitoring. In secondary implants for aphakia—especially iris-fixated lenses like Artisan (Ophtec BV, Groningen, The Netherlands) and sutureless scleral-fixated lenses such as the Carlevale IOL (Soleko, Rome, Italy)—or those implanted via the Yamane technique, OCT provides high-resolution visualization of haptic fixation, IOL stability, and potential complications, including tilt or decentration. This review also highlights comparative insights between fixation techniques, underscores the need for standardized OCT protocols, and discusses the integration of artificial intelligence tools. In summary, the routine use of OCT in the preoperative and postoperative management of phakic and secondary IOLs has been increasingly incorporated into clinical practice, as it enhances clinical decision-making and improves patient outcomes.

## 1. Introduction

Phakic intraocular lenses (pIOLs) and secondary intraocular lens (IOL) fixation techniques have expanded the options for refractive and cataract surgery, while introducing unique anatomical considerations that require high-resolution imaging for optimal outcomes [1,2,3,4,5].

PIOLs—including posterior chamber implantable collamer lenses (ICL, STAAR Surgical AG, Nidau, Switzerland) and anterior chamber iris-claw (e.g., Artisan/Artiflex, Ophtec BV, Groningen, The Netherlands)—are implanted without removing the natural crystalline lens, often in young patients with high refractive errors. Secondary IOL fixation refers to IOL implantation in eyes without capsular support (e.g., after complicated cataract surgery or lens dislocation), using techniques such as transscleral suture fixation, flanged intrascleral haptic fixation (Yamane technique), sutureless scleral plug lenses (Carlevale lens, Soleko SpA, Rome, Italy), or prepupillary or retropupillary iris-claw fixated lenses. These advanced IOL procedures present challenges in sizing, positioning, and long-term safety—areas where optical coherence tomography (OCT) has emerged as a critical tool [1,6,7,8].

In recent years, anterior segment OCT technology has advanced from qualitative imaging to enable precise quantitative analysis of key parameters, attracting increasing attention in IOL management. Table 1 offers a comprehensive overview of the various OCT models currently available on the market, highlighting their key features, specifications, and technological innovations.

Previous studies, including those from our group, laid the groundwork for understanding the critical anatomical parameters that influence IOL performance. This research demonstrated how OCT can provide high-resolution measurements of vault height, anterior chamber depth (ACD), iridocorneal angle metrics, lens tilt and decentration, and crystalline lens rise (CLR), which are critical for the selection, positioning, and follow-up of pIOLs such as ICL and Artisan lenses [4,9,10,11]. In the context of secondary IOLs, OCT enables the visualization of haptic fixation, scleral tunnel positioning, and iris configuration abnormalities such as reverse pupillary block [8]. Compared to older methods such as ultrasound biomicroscopy (UBM) or Scheimpflug imaging, modern OCT offers fast, contact-free scanning with improved depth penetration and automatic measurements [1,12].

Building upon these findings, this narrative review aims to expand the scope of anterior segment OCT’s clinical applications, focusing specifically on phakic and secondary IOLs. We compile current clinical evidence—from imaging studies and large patient cohorts to emerging artificial intelligence (AI) applications—to show how OCT enhances the visualization of IOL position, aids in detecting early complications, supports lens sizing and surgical planning, and quantitatively monitors outcomes. Key imaging metrics, such as vault distances, anterior chamber angles, IOL tilt/decentration, and their clinical significance, are covered, along with safety thresholds based on the literature. We also compare OCT with UBM for specific parameters and examine recent advances, including swept-source OCT and AI-driven analysis. Limitations and future directions are addressed, noting that although OCT has greatly improved the management of these lenses, further innovations and long-term data are needed for newer techniques. Overall, our goal is to demonstrate that, when used correctly, OCT significantly enhances the safety and effectiveness of both phakic and secondary IOL procedures, guiding ophthalmologists from preoperative screening to long-term follow-up [1].

### Search Strategy and Selection Criteria

We conducted a comprehensive literature search to identify all relevant studies on the clinical applications of anterior segment optical coherence tomography (AS-OCT) in managing phakic and secondary intraocular lenses (IOLs). The main databases searched included PubMed/MEDLINE, Embase, and the Cochrane Library.

The search was restricted to studies published between 1 January 2005, and 30 May 2025, to ensure the inclusion of contemporary data and surgical techniques. Only human studies published in English were considered. Core search terms included combinations of: “anterior segment optical coherence tomography,” “OCT,” “phakic intraocular lens,” “ICL,” “iris-claw lens,” “secondary intraocular lens,” “scleral fixation,” “Yamane technique,” “Carlevale lens,” “vault,” “tilt,” “centration,” and “artificial intelligence.”

Inclusion criteria were: (1) peer-reviewed publications reporting the use of AS-OCT for evaluation, planning, or follow-up of phakic or secondary IOLs; (2) original articles (prospective or retrospective), case series, randomized trials, systematic reviews, meta-analyses, and consensus guidelines; and (3) articles providing clinically relevant data on OCT-based measurements, complications, or outcomes. Exclusion criteria were: conference abstracts without full text, non-peer-reviewed material, animal studies, and purely technical reports without clinical application.

Study selection was performed independently by two authors who reviewed titles and abstracts, followed by full-text evaluation of potentially eligible studies. While no formal PRISMA protocol was registered, we emphasized methodological rigor in selecting the most relevant and clinically informative publications. The evidence base primarily consists of observational studies, case series, and expert consensus, with a limited number of randomized trials; these limitations are acknowledged in the discussion. While a structured search strategy was applied to ensure rigor and transparency, it does not follow systematic review or meta-analysis methodology, and therefore does not claim exhaustive completeness.

## 2. OCT in the Clinical Evaluation and Management of Phakic IOLs

### 2.1. Screening, Preoperative Planning, and Surgical Optimization

Robust imaging is as important before as after pIOL implantation. Preoperative anterior segment OCT helps determine a patient’s candidacy for pIOLs and guides lens selection (sizing) and surgical planning [2]. Key parameters, such as anterior chamber dimensions and angles, which are measurable via anterior segment OCT, often dictate eligibility, as pIOL candidates require sufficient ACD to protect the endothelium. For instance, many surgeons use a cutoff such as ACD (from endothelium to crystalline lens) above 2.8 mm for myopic ICL, 3.0 mm for hypermetropic ICL, and 3.0 mm for anterior chamber lens implantation. This ensures that even with an ICL in place, at least ~1.5–2.0 mm will separate it from the cornea (given the typical ICL vault of ~0.5 mm) [2,13,14].

Historically, ICL size (overall length) was chosen based on external white-to-white (WTW) and ACD. However, errors in sizing could occur because WTW is an indirect proxy for sulcus diameter. Newer generation imaging techniques (OCT or UBM) provide more direct measures of anterior chamber width—essentially the WTW internally, sometimes referred to as the angle-to-angle (ATA) distance. Studies using Visante OCT have shown that the horizontal ATA diameter has better reproducibility than external WTW and, when used in sizing formulas, can improve vault predictability [14,15]. Besides ATA, CLR (the distance the crystalline lens anterior pole protrudes beyond the plane joining the iridocorneal angles) measured by OCT was the other most influential parameter in a regression model for vault prediction [16,17]. A higher crystalline lens rise indicates a more anteriorly vaulted lens, often correlating with less vault (since the ICL has less space) [14].

Incorporating these OCT metrics, formulas such as the NK formula (Nakamura–Kojima) and others have been developed to more accurately determine ICL size [18]. Nakamura et al. introduced a sizing method where high-frequency OCT was used to measure ATA and distance between scleral spurs; their approach yielded vault predictions within ±100–150 μm in the majority of cases [1,19,20]. In clinical practice, some surgeons now obtain a swept-source OCT scan (e.g., CASIA, Tomey Corporation, Nagoya, Japan, or Anterion, Heidelberg Engineering GmbH, Heidelberg, Germany) preoperatively—which provides ACD, angle metrics, CLR, and an estimation of sulcus-to-sulcus via image or built-in algorithms—and input these into a formula or AI calculators for ICL sizing [18]. This is gradually supplanting the older reliance on manual WTW and empirical nomograms.

Beyond sizing, OCT contributes to planning the actual implantation or fixation technique. In ICL surgery, a critical aspect is determining whether a peripheral iridotomy (PI) is necessary. The latest ICL model (V4c with a KS AquaPORT central hole) is designed to obviate the need for PI; however, if preoperative OCT shows abnormally narrow angles or plateau iris anatomy, a small minority of surgeons still elect to perform a PI prophylactically to avoid pupillary block in the early postoperative period [1]. OCT of the iris configuration can identify plateau iris (characterized by a sharp peripheral iris drop-off and a closed angle, despite normal ACD) and prompt a combined approach (PI or iridoplasty, along with ICL) [21].

For iris-fixated phakic lenses (Artisan/Artiflex), OCT can also help ensure the eye has sufficient irido-corneal anatomy: e.g., measuring central ACD and peripheral iris contour. If the anterior chamber is shallow or the iris has an abnormal shape, an iris-claw lens may not be safe. Some surgeons also use OCT to check iris thickness and morphology (to predict ease of enclavation and risk of iris stress and pigment dispersion syndrome). Additionally, OCT can simulate how the lens will sit: some software allows overlay of a lens model on the cross-section to visualize the expected vault and clearance.

Anterior segment OCT provides a comprehensive preoperative map: dimensions (ACD, ATA) to select lens model and size, anatomy (angle, iris, sulcus) to choose a safe technique, and anticipated fit (by simulating vault and clearance). Sizing accuracy in ICL has improved significantly; in fact, modern studies using OCT-informed formulas report over 90% of eyes achieving vault within ±0.2 mm of target [18].

### 2.2. Intraoperative Guidance for pIOL

The role of intraoperative optical coherence tomography (iOCT) in ICL surgery has become increasingly relevant, providing real-time, high-resolution visualization of the ICL unfolding, position, vault, and its interaction with ocular structures.

Numerous studies have demonstrated a strong correlation between intraoperative and postoperative vault measurements, with correlation coefficients as high as *r* = 0.969 on day 1 and *r* = 0.945 at one month, confirming the predictive value of iOCT in surgical planning and postoperative outcomes [22]. In a large prospective study of 574 eyes, intraoperative vault measurements also showed a strong correlation with vault at 4 and 24 h postoperatively (*r* = 0.85 and *r* = 0.745, respectively), highlighting iOCT’s utility in immediate postoperative prediction [23].

Beyond measurement, iOCT supports intraoperative decision-making. In a prospective case series of 25 eyes, iOCT-guided rotation of the ICL in situ reduced excessive vault from a mean of 1148 μm to 740 μm (a reduction of 408 μm, *p* < 0.001), achieving more favorable vault outcomes postoperatively [24].

Importantly, iOCT enables early detection of complications such as lens-crystalline contact, misorientation (e.g., upside-down ICL), or extremely low or high vaults—allowing for immediate corrective actions such as lens repositioning or exchange before concluding surgery, this being particularly useful in eyes with atypical anatomy. This reduces the likelihood of postoperative complications such as anterior subcapsular cataract, pupillary block, or angle-closure glaucoma [25].

### 2.3. Postoperative Measurements

#### 2.3.1. Vault Measurement

**Figure 1:**
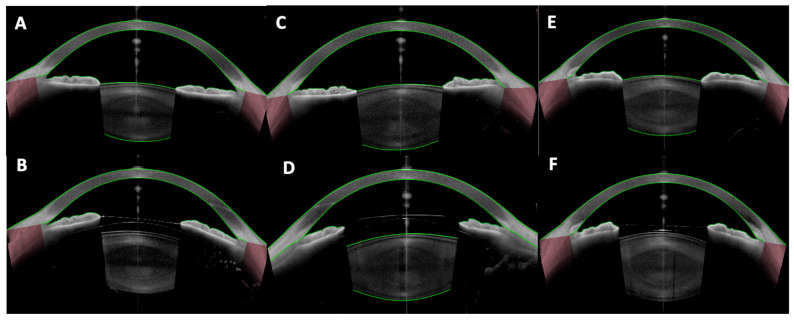
Vault assessment and angle configuration in phakic IOLs. (**A**,**B**) Preoperative anterior segment optical coherence tomography (AS-OCT) and optimal vault (~500 µm) after implantable Collamer lens (ICL) implantation, respectively; (**C**,**D**) Preoperative AS-OCT and high vault (>1000 µm) after ICL implantation, respectively; (**E**,**F**) Preoperative AS-OCT and low vault after ICL implantation (<250 µm), respectively.

One of the foremost uses of anterior segment OCT is to measure the central vault of posterior chamber pIOLs like the ICL (Figure 1). The vault is defined as the perpendicular distance between the posterior pIOL surface and the anterior crystalline lens capsule. A widely cited target considered ideal is a vault of approximately 250–750 μm in normal lighting conditions. In a properly sized ICL, the vault remains sufficient even under physiological changes [17,18,26,27]. Normative vault ranges have been established in large studies using OCT and reporting an average vault of around 500–600 μm with standard deviations of ~200 μm [28,29,30,31]. 

Postoperative vault is critical to minimize complications. Approximately 5–10% of eyes may end up with low (<250 μm) or high (>1000 μm) vaults even with formula-based sizing [14]. OCT can detect scenarios like an oversized ICL causing iris convexity (iris pushed forward by a high vault) or an undersized ICL that is “collapsing” onto the crystalline lens. It is indispensable to identify these outliers early so that prophylactic measures can be taken (e.g., prophylactic iridotomy for a high vault to prevent block, or ICL exchange for a very low vault to prevent cataract) [17].

If the vault is too low, for example, due to a severely undersized or dislocated ICL, it may come into direct contact with the crystalline lens—something that can precipitate an anterior subcapsular cataract often before it is visible clinically [32]. OCT might reveal focal obliteration of the space between ICL and crystalline lens, or a hyperreflective change on the anterior crystalline lens capsule suggestive of early cataractous change [33]. In extreme cases, an OCT scan may directly show lens contact. In extreme cases, long-term ICL–crystalline lens contact can occur, leading to cataract formation; such complications have been documented in clinical series, where OCT findings and clinical progression prompted ICL explantation [32]. On the other hand, if the vault is excessive, it can crowd the anterior chamber, elevating intraocular pressure or causing angle closure or iris pigment dispersion, as well as alterations in pupillary dynamics that could lead to focusing difficulties and halos [34,35].

Several studies have validated OCT for measuring the vault. Almorín-Fernández-Vigo et al. [28] reported that anterior segment OCT (Optovue RTVue, Fremont, CA, USA) measured ICL vault with excellent intra- and interobserver reproducibility (intraclass correlation > 0.94). In their series of 80 eyes, the mean OCT-measured vault was ~559 μm, and OCT tended to report vault values slightly higher than those reported by Scheimpflug tomography (Pentacam, Oculus Optikgeräte GmbH, Wetzlar, Germany). On average, OCT vault readings were ~128 μm greater than Pentacam measurements (558.8 vs. 430.1 μm). This systematic offset has been echoed in other comparisons and with UBM: Zhang et al. found central vault by OCT to be significantly higher than by UBM (0.50 vs. 0.44 mm on average) in post-ICL eyes [27]. Similarly, Wan et al. [36] noted that OCT showed higher ACD, distance from the corneal endothelium to the ICL and central vault measurements than both Pentacam and UBM, while Pentacam showed lower measurements than UBM. Nonetheless, the agreement is high (Pearson r ~0.88–0.89 between OCT and UBM vaults) and both methods show excellent repeatability. However, it is recommended to use the same device longitudinally for consistency [27]. Therefore, OCT is a reliable tool for vault measurement, but clinicians should be aware of calibration differences—vault criteria derived from OCT (e.g., ideal 500 μm) may not directly translate to Scheimpflug or UBM values [1,14].

#### 2.3.2. Phakic Lens Position

OCT also visualizes ICL centration and distance to surrounding structures. High-resolution cross-sections not only show the pIOL’s vault centrally, but can also detect if the lens is vaulting unevenly (tilted) or touching the crystalline lens or iris at any point, most often at the level of the iris sphincter. Similarly, OCT can show decentration or disenclavation (one haptic slipping from the iris) of iris-fixated lenses. Calzetti et al. [6] used CASIA OCT to measure this, with anterior iris-claw lenses showing median tilt of ~5.6° and decentration of ~0.24 mm.

Pigment dispersion syndrome, characterized by pigment accumulation on the corneal endothelium and in the anterior chamber angle, is a subtle but significant complication after pIOL implantation and is typically caused by iris chafing. While OCT cannot visualize pigment granules directly, it is instrumental in identifying the anatomical causes of dispersion. For instance, an excessively high vault (>1000 μm) in an ICL may cause the posterior iris to bow forward and make contact with the crystalline lens at the mid-periphery—clearly visible on angle OCT scans. Similarly, an anterior chamber pIOL positioned too close to the iris may result in constant rubbing. OCT cross-sections showing minimal space between the pIOL and the iris can raise suspicion of ongoing pigment shedding. These structural findings, when correlated with clinical signs such as transillumination defects, Krukenberg spindle, or pigment in the angle, help confirm the diagnosis. Ultimately, OCT enhances diagnostic certainty in pigment-related complications by directly revealing iris-lens interactions, such as iris concavity, pIOL tilt, or asymmetric enclavation [1,17].

#### 2.3.3. Distance to Corneal Endothelium

Anterior segment OCT provides critical information about ACD, which is important not only pre- but also post-pIOL. OCT can directly measure the space between a pIOL and the corneal endothelium, allowing clinicians to verify postoperatively whether the lens is positioned within safety zones.

Baikoff et al. first demonstrated the importance of this variable. In a 2004 study using OCT, they systematically measured distances from an iris-supported pIOL to ocular structures and proposed minimum safe separations for the cornea (≥1.5–2.0 mm) and crystalline lens (≥0.5 mm) [1,37]. Hence, if a postoperative OCT shows an iris-claw lens vaulting too close to the cornea (say 1.2 mm centrally or at the periphery), surgeons might decide to explant the pIOL proactively to prevent corneal decompensation. Similarly, a shallower chamber after an ICL (due to a thick lens or high vault) means less endothelial safety distance [37,38].

For Artisan/Artiflex lenses, studies by Doors et al. [38] used time-domain anterior segment OCT (Visante, Carl Zeiss Meditec AG, Jena, Germany) to measure both the central pIOL-to-endothelium distance and the peripheral (pIOL edge to endothelium) distance. In 242 eyes, the mean distance at the pIOL edge was ~1.37 mm (with many eyes slightly under the historically recommended 1.5 mm). Importantly, they found a statistically significant correlation between smaller edge distances and higher rates of long-term endothelial cell loss. Eyes with an edge distance of ~1.15 mm (1 SD below mean) had nearly double the annual endothelial loss (~1.8%/year) compared to those with ~1.59 mm distance (1 SD above mean, ~0.15%/year loss). These findings reinforced the empirical safety rule that one should strive for at least 1.5 mm clearance at the narrowest point between an anterior chamber pIOL and the cornea [1].

In clinical practice, specular microscopy remains the gold standard for monitoring endothelial cell density; however, OCT complements it by identifying anatomical risk factors that may accelerate cell loss.

#### 2.3.4. Angle Anatomy

**Figure 2:**
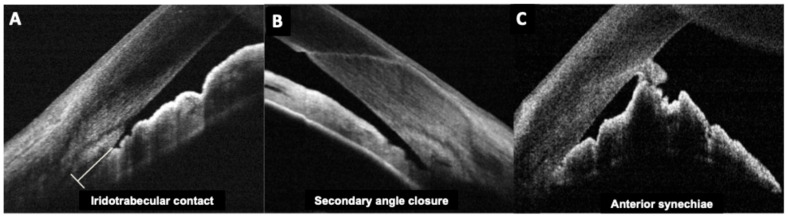
Anterior segment optical coherence tomography images of (**A**) iridotrabecular contact; (**B**) secondary angle-closure and (**C**) Anterior synechia secondary to posterior chamber intraocular lens implantation.

Fourier-domain and swept-source anterior segment OCT can image the iridocorneal angle in detail. After pIOL implantation, angle anatomy often changes: posterior chamber ICLs typically cause a mild reduction in angle width and volume because the iris is slightly pushed forward by the ICL (Figure 2). A study revealed postoperative angle opening distance decreased by ~20–25% after ICL, though the angles generally remained open in appropriately selected eyes [27]. Similarly, our group determined iridocorneal angle changes prospectively in ICL patients, indicating initial angle narrowing of 39–45% which was stable beyond 1 month postoperative [39].

On the other hand, anterior chamber pIOLs (angle-supported lenses) can cause even more pronounced angle shallowing and sometimes focal synechiae formation at the lens footplates, which can be visualized with anterior segment OCT [40,41].

Pupillary block glaucoma is a pIOL-related emergency that occurs when aqueous flow from posterior to anterior chamber is impeded, causing iris bombe (forward bowing of the iris) and acute angle closure. In pIOL patients, this can happen if the PI is blocked, if an ICL without a central port traps fluid or if pigment or viscoelastic obstructs the central port [42,43].

**Figure 3:**
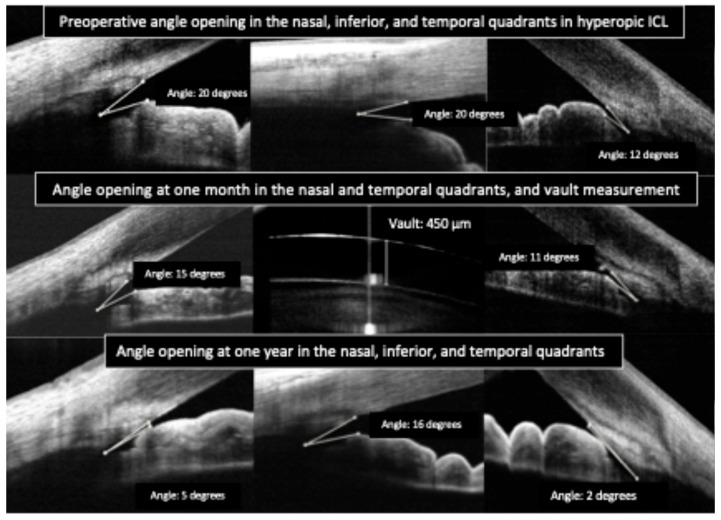
Preoperative and postoperative angle opening after hyperopic implantable Collamer lens (ICL) implantation.

**Figure 4:**
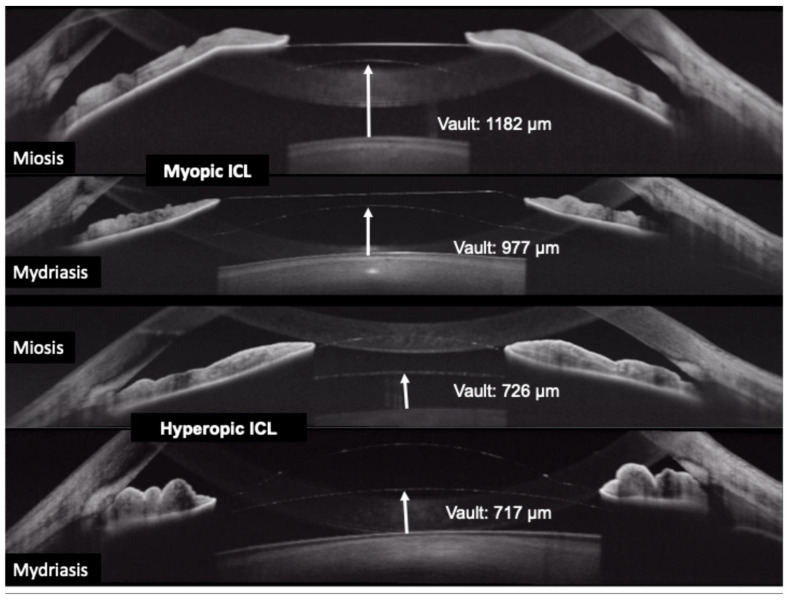
Change in the vault during miosis and mydriasis after myopic (upper rows) and hyperopic (bottom rows) implantable collamer lens (ICL) placement.

Clinicians have reported using OCT to differentiate pupillary block from other causes of high intraocular pressure. In pupillary block, the OCT shows the iris bowed forward in a classic “iris bombe” convexity contacting the lens or ICL, with a deep posterior chamber and shallow anterior chamber. Anterior segment OCT can confirm iris bombe configuration, even when a hazy cornea or a small pupil limits the gonioscopic view [44]. Anterior segment OCT allows for a precise evaluation of hyperopic ICL cases, which usually present with narrower baseline angles, as well as for monitoring the dynamic changes in vault and angle configuration that occur with miosis and mydriasis (Figure 3 and Figure 4).

### 2.4. Postoperative Monitoring and Long-Term Follow-Up

Once a phakic IOL is correctly implanted, long-term monitoring is essential to ensure it continues to perform safely. OCT has become a mainstay of pIOL follow-up visits, alongside pressure checks and endothelial cell counts [1]. Table 2 shows frequently measured OCT variables and safety thresholds in pIOLs.

After ICL implantation, the vault can evolve over time due to changes in the crystalline lens (which thickens with age) or ICL positioning shifts. Several studies have tracked vault changes over the years using OCT, and generally, the vault tends to gradually decrease with time, which could increase the risk of anterior subcapsular cataract [14,33]. This decrease in the vault correlates with slight crystalline lens growth, and patient age is inversely associated with vault [14]. In eyes that developed cataract, a precipitous drop in vault often preceded lens opacification [33]. Although angles usually stabilize after 1 month, monitoring is nevertheless mandatory [39]. Thus, periodic OCT vault assessment (e.g., at 6 months, 1 year, and annually thereafter) is necessary as the eye’s anatomy changes (Table 3).

OCT is also useful for monitoring pIOL centration and tilt over time, particularly if a patient experiences visual symptoms like glare or monocular diplopia years after pIOL surgery. Although phakic IOLs are generally stable (the ICL, for example, centers well in the ciliary sulcus in most cases), subtle decentrations or tilts can emerge, especially if ocular anatomy changes (e.g., ciliary body atrophy, trauma, iris cysts). Even a moderate tilt can induce higher-order aberrations (particularly coma) and reduce the quality of vision, as Cui et al. [45] highlighted in a study with secondary scleral-fixated IOLs. Although that study concerned secondary IOLs, the principle holds—decentered optics degrade vision, and OCT can detect it objectively.

For anterior chamber pIOLs, peripheral anterior synechiae (PAS) and chronic angle closure are late complications leading to glaucoma. Gonioscopy can identify PAS, but OCT can map the extent of angle closure around 360 degrees more systematically. Visante OCT studies showed that after angle-supported PIOL implantation, a significant number of eyes develop some PAS, which can be visualized as peripheral iris tissue fused to the cornea with no space on OCT scans. Monitoring these findings can guide timely intervention (e.g., removing the pIOL if progressive PAS threatens extensive angle closure). Similarly, periodic OCT of the cornea-pIOL distance can reveal a progressive decrease, indicating endothelial threat.

**Figure 5:**
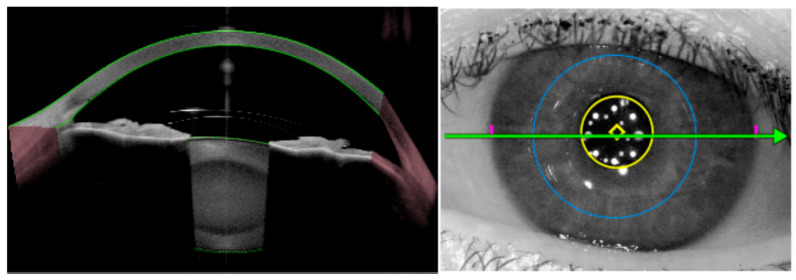
Anterior iris-fixated intraocular lens (Artiflex) in a phakic patient, visualized by AS-OCT. Good centration of the lens is observed, as well as its spatial relationship with surrounding ocular structures.

Anterior segment OCT is also useful for the evaluation of anterior iris-fixated intraocular lenses, allowing detailed assessment of lens position and the distance from the corneal endothelium (Figure 5).

### 2.5. Planning for pIOL Exchange or Reposition

While most phakic IOL implantations are successful, some cases may benefit from postoperative adjustments, for which OCT proves to be a valuable tool in planning and optimizing outcomes.

If an OCT at 1 month shows a very low vault (for example, 50 μm with ICL nearly touching the lens), the surgeon may plan an ICL exchange for a larger size. OCT can guide the timing and lens choice: for instance, if a 0.5 mm larger ICL was initially used, reducing by 0.5 mm might be advised to lower the vault height. Conversely, if the vault is extremely high (>1200 μm) and the patient has elevated IOP or shallow angles, planning to exchange it for a smaller ICL or rotate it from a horizontal to a vertical position can help decrease the vault. In both cases, OCT measurements provide objective evidence to justify reoperation and serve as a baseline for comparison after the exchange.

When repositioning an iris-claw lens that has tilted or a haptic that has partially disengaged, OCT can pinpoint the degree of misalignment (e.g., 20° tilt). This informs the surgical approach—the surgeon knows which haptics to release and re-enclavate to balance the lens. For sutured scleral-fixated IOLs that decenter (possibly due to suture loosening), OCT can quantify decentration (e.g., 1 mm downwards). This data supports the need for a resuture or adjustment.

## 3. OCT for Secondary (Pseudophakic) IOL Fixation Techniques

**Figure 6:**
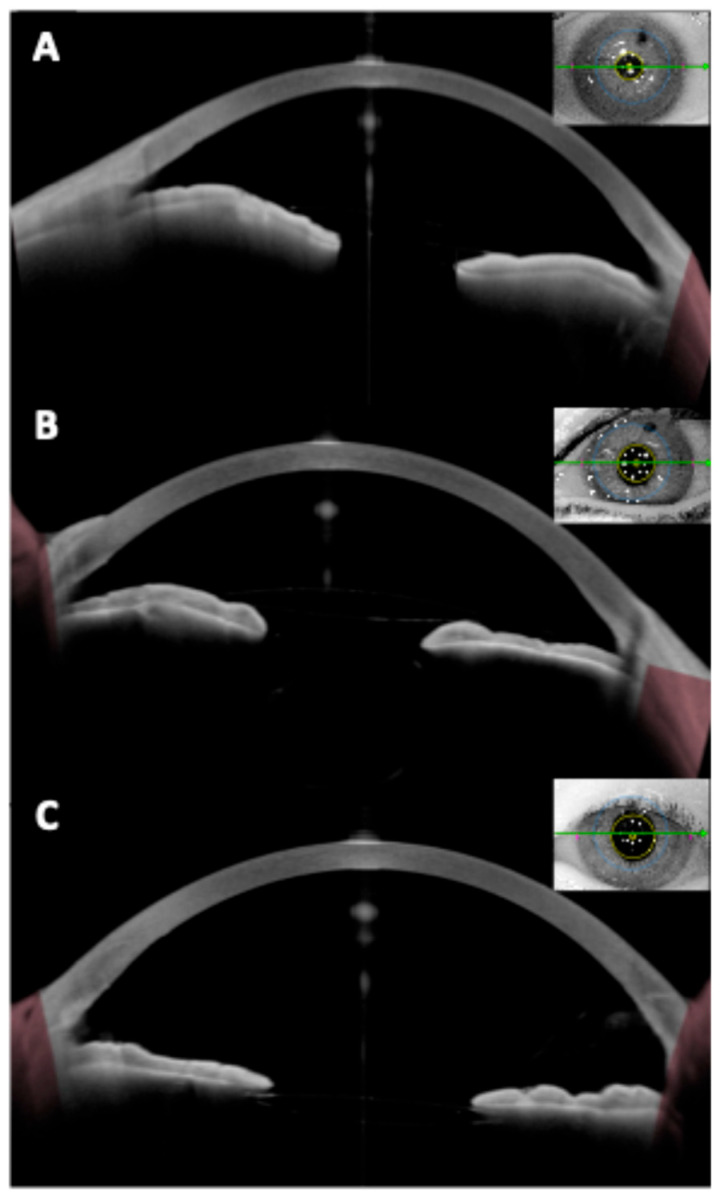
(**A**,**B**) Images showing an anterior iris-fixated intraocular lens (Artisan) implanted in an aphakic eye, with good optic centration. (**C**) Image of a posterior (retroiridian) iris-fixated intraocular lens, also implanted in an aphakic eye, demonstrating proper centration.

**Figure 7:**
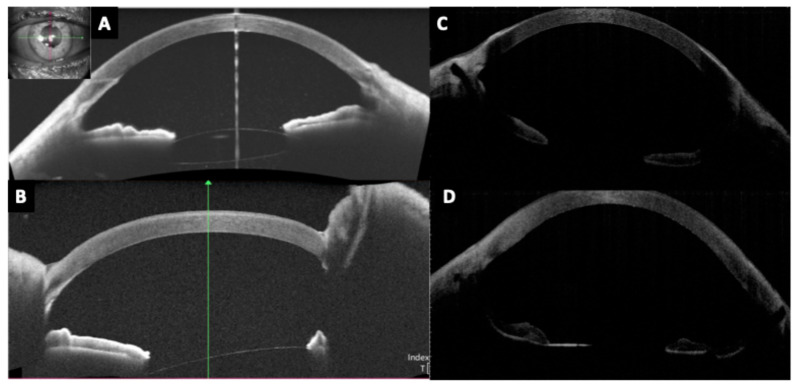
Anterior segment optical coherence tomography images showing a well-positioned Carlevale sutureless scleral-fixated intraocular lens with (**A**) vertical and (**B**) horizontal cross-sectional scans demonstrating central optic positioning, slight tilt, and adequate distance from the corneal endothelium and iris plane. (**C**,**D**) Images show both haptics of the Carlevale lens anchored within intrascleral tunnels approximately 1.5–2.0 mm posterior to the limbus.

Beyond phakic refractive lenses, OCT has significant utility in secondary IOL fixations—cases where an intraocular lens is implanted in an eye without capsular support. This includes scleral-fixated IOLs (both sutured and sutureless Yamane-type), Carlevale lenses (a one-piece lens with T-shaped scleral anchoring plugs), iris-claw lenses used for aphakia, and other innovative techniques [46,47,48,49,50] (Figure 6 and Figure 7). These scenarios present unique imaging challenges, as the IOL is often located in the posterior chamber or at the iris plane, and the supports (haptics, flanges, or sutures) are not within the visible pupil.

Anterior segment OCT (especially swept-source) and even posterior segment OCT (for certain views) have been employed to evaluate the positioning and outcomes of these secondary IOLs (Table 3). OCT assures proper lens positioning (centration/tilt), verifies fixation integrity (flange/plug location), and detects any secondary anatomical changes (iris configuration, macular edema) that might require intervention. This has translated into improved patient outcomes. For example, the knowledge that Yamane and sutured fixations yield comparable tilt means surgeons can choose either technique based on other factors [45]. The ability to detect issues like reverse pupillary block (RPB) early via OCT has led to modifications in technique (such as performing an iridotomy at the time of Carlevale lens insertion) [51]. As new secondary lens designs emerge, OCT will likewise be critical in evaluating their safety.

### 3.1. Preoperative Planning

When planning secondary IOL fixation (aphakic eyes), anterior segment imaging is crucial to choose the appropriate method as it can measure sulcus anatomy and scleral thickness where flanges would be fixated [11,52]. For example, the Yamane intrascleral fixation involves creating scleral tunnels ~2 mm posterior to the limbus. High-resolution OCT of the pars plana/sclera can help gauge scleral thickness to ensure the tunnels will securely cover the haptics. If a patient has very thin sclera (e.g., pathological myopia or previous surgery), one might favor a sutured technique over Yamane. Conversely, for the Carlevale lens, the plugs reside in scleral pockets at ~3 mm from limbus; OCT or UBM can measure the distance between opposite iris root areas or sulci to ensure the one-size Carlevale (13.2 mm length) will fit without excess slack or stretch [53]. Generally, eyes with a very large ciliary ring (>13 mm sulcus) might be better served by a custom sulcus-fixated IOL with sutures, whereas moderate sizes can take Carlevale. These decisions are aided by imaging the horizontal sulcus-to-sulcus distance. While UBM is the classic for sulcus measurement, some OCT devices (like Visante) can image the angle recess and give an indirect estimate of sulcus size (though the actual ciliary sulcus is behind the iris). In one study, a regression using OCT-measured ATA was actually a better predictor of vault than using WTW, supporting the idea that OCT captures more of the internal anatomy [1].

### 3.2. Tilt and Decentration in Scleral-Fixated IOLs

Tilt and decentration are particularly pertinent for secondary IOLs because the lack of capsular bag support can lead to more IOL movement. Several studies using OCT have quantified this. Cui et al. [45] performed a prospective comparison of Yamane flanged intrascleral fixation and traditional sutured scleral fixation, measuring IOL tilt and decentration at 3 months with CASIA OCT. They found no significant difference between the two methods: both had mean tilt ~2–3° horizontally and ~3–4° vertically, and mean decentration around 0.3 mm, with no statistically significant disparities. These values are only slightly above in-the-bag IOL norms, indicating that with proper technique, secondary fixations can achieve near-normal alignment. Similarly, Do et al. [54] found no differences using CASIA2 OCT.

Earlier studies using Scheimpflug photography were less accurate and had conflicting findings. Sül et al. [55] reported with Scheimpflug that flanged IOLs had on average ~1° less tilt than sutured. OCT being a direct measure, it most likely provides the more reliable answer: modern flanged techniques do not inherently introduce more tilt/decentration than suture fixations.

Nonetheless, individual cases can have significant tilt, and OCT can identify those outliers. For example, if a haptic is not well fixated in the scleral tunnel, the IOL might tilt towards that side. A visually significant cutoff is often ~5° tilt [53]. OCT can directly confirm if an eye exceeds that, and one may then consider re-fixating the lens. Decentration in scleral-fixated lenses tends to be directional: one study found most sutured IOLs decentered infero-nasally (due to loop slack), whereas flanged IOLs were more randomly distributed [45].

CASIA OCT polar plots may be helpful in visualizing this decentration pattern. Knowing the magnitude and direction of malposition via OCT is extremely helpful if surgical adjustment is planned—the surgeon knows which scleral fixation point to tighten or whether the IOL might need to be completely refixated [6].

### 3.3. Visualization of Scleral Fixation Points

A unique use of OCT in these cases is to visualize the scleral tunnels and haptics/flanges. Swept-source anterior segment OCT can often penetrate enough through the sclera to see a high-reflectivity foreign material (like a haptic or flange) within.

**Figure 8:**
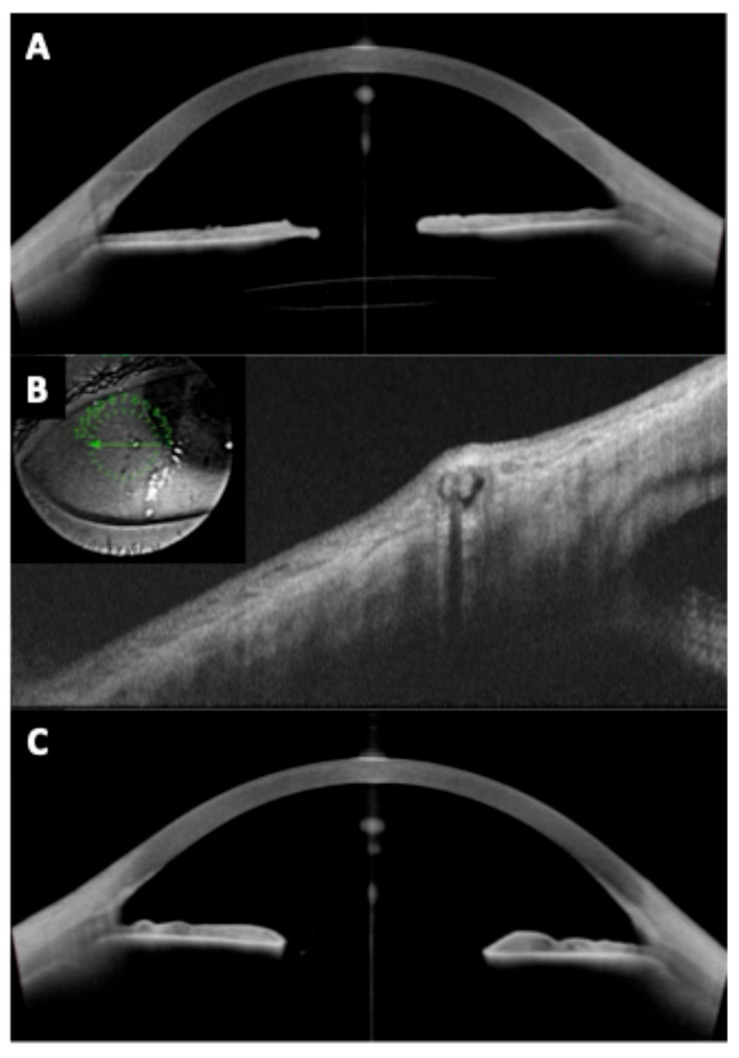
(**A**) Secondary intraocular lens (IOL) implantation using the Yamane technique. Good centration of the IOL is observed, with no significant tilt and adequate stability as confirmed by OCT. The optic is visible, although the haptics cannot be identified. (**B**) The intra-scleral and subconjunctival haptic button of the three-piece IOL is visible. (**C**) Contralateral eye of the same patient. The iris is visible on OCT, but aphakia is present due to blunt trauma that resulted in vitreous dislocation of the capsular bag–IOL complex.

For the Yamane technique, authors have shown OCT images confirming the haptic’s bulb (flange) is seated within the scleral wall at the intended depth [45]. Figure 8 shows an OCT cross-section at a Yamane flange site, where the hyperreflective tip of the haptic is visible within the sclera (between the outer scleral surface and the tunnel floor), with no extrusion, confirming correct placement [45]. If, instead, the flange was too superficial, OCT might show it tenting up the sclera or even eroding through (a break in the scleral contour). Such findings on early postoperative OCT could prompt reinforcing that area (with graft or suturing) before a frank exposure occurs.

Regarding the Carlevale lens, which features a T-plug at each haptic end, OCT can similarly confirm the position of the plug under the partial-thickness scleral flap [53]. A study by Rouhette et al. [56] on Carlevale outcomes noted stable IOL positioning with good centration; imaging was used to ensure the T-plugs were well covered by scleral tissue in all cases. If a plug were partially exposed, one would see it directly abutting the conjunctiva on OCT.

### 3.4. Position of Iris-Claw Aphakic Lenses

Similarly, OCT can show decentration, tilt or disenclavation (one haptic slipping from the iris) of iris-fixated lenses. Calzetti et al. [6] demonstrated that CASIA OCT could detect cases where a posterior iris-claw IOL was decentered by >0.5 mm—sometimes attributable to asymmetric iris clawing—even if this was not obvious on external exam. Anterior iris-claw lenses showed median tilt ~5.6° and decentration ~0.24 mm, whereas retropupillary (posterior) iris-claw lenses had similar tilt (~5.0°) but notably larger decentration (median 0.67 mm, significantly larger than in-the-bag lenses at ~0.24 mm). Tilt direction was mainly temporal, while decentration was inferior-temporal with posterior iris-claw IOLs and scattered with anterior iris-claw IOLs.

If an iris-fixated lens is not well centered (perhaps enclavated slightly off center), OCT follow-up can document that and ensure no progressive shift. Unlike scleral fixations, iris-claw IOLs are less likely to move once healed unless one claw disengages due to trauma. If a disengagement happens, the IOL typically tilts dramatically. In this case, OCT can confirm that one haptic is free, and surgical re-enclavation would be required.

### 3.5. Intraoperative Guidance

The use of iOCT has been described to check that the IOL haptics are positioned correctly in the scleral tunnels and that the lens optic is centered at the end of surgery [7]. For example, if iOCT showed one haptic not sufficiently buried, the surgeon can re-feed it into the tunnel on the spot. IOCT is particularly useful in cases with poor visibility (corneal edema or hemorrhage)—the infrared OCT can still visualize the IOL and ocular structures. A clinical study on intraoperative OCT for IOL tilt found that surgeons could detect and correct misalignment of scleral-fixated IOLs in real time, leading to better postoperative centration [7]. Although not yet standard in routine pIOL insertion, this technology may gain utility for secondary fixations and complex scenarios.

### 3.6. Reverse Pupillary Block

A recently described issue with sutureless scleral lenses (like Carlevale or Yamane) is RPB—where the iris is pulled posteriorly against the lens optic, causing angle shallowing despite a deep overall chamber [51]. This occurs due to the closed iris and the IOL acting like a one-way valve, often exacerbated by anterior vitreous face blocking forward fluid movement. Clinically, these patients present with elevated IOP and a deep anterior chamber centrally but shallow peripherally, sometimes with iris flutter.

Anterior segment OCT is extremely useful in diagnosing RPB: it shows a gap between iris and cornea (deep anterior chamber) centrally, but iris concavity (bowing backward) and peripheral iris-cornea touch, distinguishing it from typical block (which has iris convex forward). Sánchez-Vela et al. [51] studied RPB after Carlevale lenses and used swept-source OCT (Heidelberg Anterion) to assess iris configuration before and after YAG iridotomy. OCT confirmed that the iris was vaulting backward and occluding the pupil area; after iridotomy, the iris returned to normal contour and the problem resolved. They reported RPB in ~15% of Carlevale cases, recommending a prophylactic intraoperative PI. This insight was possible through imaging and careful follow-up. Thus, OCT helps not only in observing such phenomena but also in guiding prevention strategies (e.g., noticing mild iris reverse bowing on day 1 might prompt a PI even before pressures spike).

For clarity, Table 4 summarizes the main OCT parameters associated with complication risk, their clinical implications, and the supporting evidence base, serving as a practical decision-making guide.

## 4. Emerging Trends: AI Integration and Advanced Imaging

Advances in computational analysis are now intersecting with OCT imaging of IOLs, promising enhanced predictive and diagnostic capabilities. AI and machine learning techniques have been applied both to preoperative biometry for vault prediction in ICL and to postoperative OCT images for automated analysis in pIOL and secondary IOLs.

### 4.1. AI for Vault Prediction and IOL Sizing in ICL

Predicting the postoperative vault of a phakic ICL is a complex, non-linear problem influenced by multiple biometric factors [18]. Traditional linear formulas (e.g., those provided by manufacturers or early regression models like the NK formula [19]) use a few inputs such as WTW and ACD. Recently, machine learning approaches have substantially improved vault prediction by incorporating a broader range of parameters and complex interactions [18].

Studies by Kamiya et al. [9] and Kang et al. [57] were among the first to show that supervised machine learning using anterior segment OCT metrics could outperform standard formulas in vault prediction. More recently, Chen et al. [18] published a large-scale machine learning analysis of 1941 eyes using data from multiple devices to predict vault and select ICL size. They tested various algorithms and noted that a combination of Scheimpflug, OCT and UBM data yielded the highest accuracy. Notably, in their model the sulcus-to-sulcus distance from UBM was one of the top predictors for vault and ideal size, underlining that direct sulcus measurement (something OCT cannot yet fully do) still holds value. However, they also described that even using only OCT-based parameters, the machine learning approach could achieve useful accuracy—and that an all-UBM approach could also be excellent.

Beyond sizing, AI has been explored for risk prediction: e.g., identifying which eyes might end up with suboptimal vault or complications. Cerpa-Manito et al.’s [14] work hints at multivariate risk factors for high vs. low vault (ICL compression, lens rise, myopic power, age)– an AI could combine those into a risk score. Another example is pigment dispersion risk after ICL—presumably, an algorithm could classify anterior chamber shapes on OCT to flag those likely to have iris contact.

In practical terms, surgeons might soon rely on an algorithm that inputs the patient’s OCT scans and outputs the recommended ICL size with estimated vault distribution. Indeed, a recent “big-data and AI-assisted” platform in China (using >7000 eyes) demonstrated improved accuracy of ICL sizing and is being integrated into clinical workflows [58]. Such AI models can reduce the incidence of extreme vaults and cut the need for ICL exchanges significantly. It is noteworthy that these models often incorporate both OCT and optical biometer data (keratometry, axial length, lens thickness) for a holistic prediction [1].

### 4.2. Automated OCT Image Analysis

On the postoperative side, deep learning algorithms have been developed to interpret anterior segment OCT scans. Shen et al. [58] demonstrated a deep learning system that automatically segments key structures (cornea, ICL, crystalline lens, angle points) on OCT images and then computes vault, ACD, and other parameters [59]. The system could identify the ICL and lens with high Dice similarity (~0.97) and measure vault within a mean error of only a few microns compared to experts. This removes observer variability and outputs the vault measurement compared to previous visits. Another research team published an algorithm that detects IOL tilt and decentration on CASIA OCT scans fully automatically—beneficial for evaluating large trial datasets or screening for misalignments in follow-ups [6].

These developments portend a future where AI co-pilot systems constantly monitor OCT results and alert clinicians to potential issues (e.g., “Vault has dropped by 120 μm since last year” or “Lens decentered nasally by 0.3 mm, check haptic.”). Early steps in this direction are seen in AI that predict endothelial cell loss given OCT-measured distances and time—building on works like Doors et al. [38], but with individualized projections. AI has also been leveraged for decision support: for instance, recommending when an intervention is needed. A hypothetical model could combine a patient’s vault trend, endothelial count trend, and OCT-detected iris behavior to suggest “explant now” or “safe to observe.”

### 4.3. Enhanced Imaging Modalities

While not AI, it is worth noting new OCT hardware advances that complement this field. Swept-source OCT with longer wavelengths has already improved penetration, allowing visualization of structures like the ciliary body and parts of the haptic [1]. There are prototypes of 360° anterior segment OCT scanners that can image the entire sulcus circumference. One such device is the ArcScan Insight (very high-frequency ultrasound), which can map sulcus 360°, but similar capabilities are being sought in OCT. If successful, an OCT could directly measure the sulcus diameter at any angle, further refining pIOL sizing.

Wide-angle OCT is another innovation—capturing from cornea to posterior lens in one scan. This is useful in pIOL imaging to ensure the whole lens is in frame including footplates or haptics. The Eyestar 900 and IOLMaster 700 are commercial devices combining swept-source OCT biometry with corneal topography that can measure anterior chamber, lens thickness, etc., and even attempt to measure vault of a phakic IOL automatically [60]. Such multi-functional devices will likely become standard, streamlining follow-ups—e.g., one scan yields refraction-related data and vault/angle info.

Furthermore, anterior segment dynamic OCT allows video-rate imaging of anterior segment structures with high-speed swept-source systems like the CASIA2, examining how the ICL vault or iris shape changes with accommodation or varying lighting [61]. Kato et al. [62] demonstrated that vault significantly decreased during photopic conditions (476.1 ± 219.6 μm compared to 521.1 ± 220.4 μm under scotopic conditions, *p* < 0.001) and during accommodation (454.8 ± 224.9 vs. 481.6 ± 219.1 μm, *p* < 0.001), likely due to changes in iris-lens interaction. These dynamic changes are clinically relevant. An excessive decrease in vault during accommodation might predispose to lens touch or pigment dispersion, while increased vault in darkness could affect aqueous dynamics or angle width. AI-based analysis could automatically detect abnormal vault behaviors, leading to personalized risk profiles for complications such as cataract formation or angle narrowing. González-López et al. [63] further corroborated these findings, reporting that dynamic vault changes occur in response to both light and accommodative stimuli, and that these variations are significantly influenced by ICL size and baseline vault. Their study reinforces the importance of individualized ICL sizing and suggests that dynamic anterior segment assessment could help identify eyes at greater risk of mechanical complications related to excessive vault fluctuation. Despite the promising insights provided by dynamic OCT, current evidence stems mainly from small sample sizes and short follow-up periods. The extent to which dynamic changes in vault or angle anatomy predict long-term safety outcomes remains uncertain, and further studies with larger and more diverse cohorts are needed to establish clinical applicability.

## 5. Limitations and Future Directions of OCT in Phakic and Secondary IOL Management

Despite its transformative role, the use of anterior segment OCT in the management of phakic and secondary IOLs is not without limitations. One important consideration is line-of-sight dependency and anatomic shadowing. Non-contact OCT requires a clear optical path, which may be compromised in cases of corneal opacities, dense iris pigmentation, or very narrow angles. In such settings, visualization of posterior chamber structures—particularly the ciliary sulcus or scleral haptics—can be incomplete. UBM remains superior for imaging sulcus-to-sulcus distance, and is still recommended in borderline anatomical cases or eyes with atypical iris anatomy [18].

Another relevant issue is the variability across OCT platforms. Measurements such as vault or ACD may differ between time-domain, spectral-domain, and swept-source OCT systems. These discrepancies arise not only from technical resolution but also from differing anatomical definitions (e.g., epithelium- vs. endothelium-based ACD). Therefore, clinicians should interpret safety thresholds (e.g., vault limits) in the context of the specific device used, and ideally monitor trends with the same system throughout follow-up [28].

Image interpretation and measurement reproducibility also pose challenges. Although OCT imaging is generally intuitive, artifacts such as mirror images, signal doubling, or poor boundary detection can lead to misinterpretation. Technician training and clinician experience are essential, especially when measuring vault or identifying IOL tilt and decentration. Interobserver reproducibility is generally high (e.g., ICC > 0.9 for vault), but slight variability may occur in borderline measurements or low-quality scans. Standardized measurement protocols help mitigate these discrepancies. Nonetheless, the learning curve associated with advanced OCT interpretation should not be underestimated. Recognizing subtle signs of reverse pupillary block, iris chafing, or early tilt may take time and experience.

There is also a lack of large-scale normative data for newer techniques, particularly secondary IOLs like Yamane or Carlevale lenses. While early studies suggest average tilts around 3–4°, long-term OCT-based evaluations of flange position, erosion, or lens stability remain limited. Until larger cohorts confirm acceptable variability ranges, minor asymmetries or misalignments should be interpreted cautiously [45].

Emerging AI tools and predictive models are promising but still face issues of generalizability. Most existing vault prediction models have been trained on Asian populations, where the anatomy of the anterior chamber differs from that of Caucasian cohorts. Factors such as lens thickness, iris configuration, and sulcus diameter may affect model accuracy across ethnic groups. Therefore, external validation across diverse populations is necessary before these algorithms can be universally adopted. Furthermore, many of these models remain retrospective and lack prospective validation in large, multicenter trials. Broader validation or retraining on diverse datasets is required before widespread adoption [1].

Practical limitations must also be acknowledged. Implementation of AS-OCT in resource-constrained settings presents unique challenges, mainly due to high acquisition costs and limited availability of advanced platforms. In these contexts, OCT may need to be prioritized for patients at higher risk of complications (e.g., shallow anterior chamber, borderline vault, or complex secondary IOL fixation). Portable or lower-cost OCT devices, although with reduced resolution, could still provide valuable screening information and improve safety in these cases. Importantly, several commercially available OCT models offer adequate image quality at a lower cost than the most advanced swept-source systems, and these may represent a feasible compromise for institutions with limited resources. Shared access models between institutions, integration into tertiary referral centers, and simplified imaging protocols focusing on the most critical parameters (vault, angle width, cornea–IOL distance) may further facilitate broader adoption. Teleophthalmology solutions, whereby OCT scans are acquired locally and interpreted remotely by specialists, represent another potential strategy to extend access in low-resource environments

Moreover, advanced OCT platforms and integrated AI tools are not yet universally available, creating inequities in care. Furthermore, as this is a narrative rather than a systematic review, the evidence presented should be interpreted as a synthesis of the most relevant and clinically meaningful studies.

Looking forward, several exciting directions are emerging. OCT-based IOL sizing algorithms, automated IOL centration metrics, and remote anterior segment monitoring (possibly even at-home devices) could transform how we select, implant, and follow these lenses. Future OCT studies may compare the stability of different secondary IOL fixation methods (e.g., glued IOL vs. Yamane) and explore correlations between posterior segment anatomy (like axial length) and anterior segment lens positioning.

## 6. Conclusions

OCT has become an essential tool for the safe and effective management of phakic and secondary IOLs. Its high-resolution, cross-sectional imaging enables accurate measurement of critical parameters such as vault, anterior chamber depth, angle width, and IOL tilt or decentration—both preoperatively and postoperatively. In pIOLs like the ICL and iris-fixated lenses, OCT allows surgeons to apply evidence-based safety thresholds (e.g., vault ~0.5 mm, cornea-pIOL distance ≥ 1.5 mm), detect early complications such as progressive vault reduction or anterior subcapsular opacities, and make informed decisions regarding interventions like iridotomy or lens exchange. In secondary IOL fixations, especially with scleral-fixated designs like the Yamane technique or Carlevale lens, OCT has transformed surgical planning and follow-up by visualizing haptics, scleral tunnels, and flange positions—previously inaccessible to direct inspection.

Moreover, the integration of swept-source OCT, wide-angle imaging, and AI is ushering in a new era of precision: machine learning models improve IOL sizing predictions, and automated metrics facilitate long-term monitoring. These innovations support a paradigm shift toward personalized, data-driven lens implantation, enhancing safety, reproducibility, and visual outcomes.

While anterior segment OCT is not without its limitations, it has already revolutionized the safety and precision of phakic and secondary IOL implantation and is now central to the long-term management of these patients, allowing ophthalmologists to track critical parameters and detect subtle shifts that, if unaddressed, could compromise safety or vision. Continued technological innovation and broader accessibility will further enhance its role, driving the field toward safer, more personalized, and predictable refractive solutions.

In summary, OCT connects anatomical understanding with clinical decision-making, helping to reduce the occurrence of complications such as cataract, glaucoma, and corneal decompensation. As technology advances, OCT-guided management will continue to play a crucial role in achieving optimal and predictable outcomes for patients undergoing phakic or secondary IOL procedures.

## Figures and Tables

**Table 1:** Technical and Clinical Features of Anterior Segment OCT Systems.

Model	Manufacturer	OCT Technology	Axial Resolution (µm)	Scan Speed (A-scans/s)	Anterior Segment Modes	Software Capabilities	Clinical Applications in IOLs
Anterion	Heidelberg Engineering, Heidelberg, Germany	Swept-source (1300 nm)	10	100,000	Full anterior segment, IOL visualization, axial length	Vault, ACD, angle metrics, tilt, axial length, IOL centration	Phakic IOL sizing, secondary IOL centration and tilt
CASIA2	Tomey Corporation, Nagoya, Japan	Swept-source (1310 nm)	10	50,000	360° angle analysis, IOL position, corneal/sulcus analysis	Vault, ACD, angle width, IOL tilt, corneal/sulcus overlay	ICL/Artisan evaluation, scleral-fixated IOL assessment
Triton	Topcon Corporation, Tokyo, Japan	Swept-source (1050 nm)	8	100,000	Multimodal (retina + AS), IOL vault, angle and tilt	Multiplanar view, IOL vault, iris configuration	ICL follow-up, reverse pupillary block detection
Cirrus HD-OCT	Carl Zeiss Meditec, Jena, Germany	Spectral-domain (840 nm)	5	27,000	Limited AS with add-on lens, angle depth, IOL position	Basic angle analysis, limited vault and tilt (external lens)	Basic phakic IOL monitoring, preop screening
TowardPi	TowardPi Medical, Beijing, China	Swept-source (1310 nm)	10	100,000	Wide-field AS-OCT, sulcus-to-sulcus, tilt, vault	Advanced vault measurement, angle, crystalline lens analysis	Vault prediction, sizing guidance, haptic position
Dream OCT	Ioptics/OCULUS, Wetzlar, Germany	Swept-source (1300 nm)	10	100,000	AS imaging with lens vault, iris-lens relationships	Custom reports on ICL vault and anterior chamber profile	Pre/post ICL assessment, visualization of fixation sites

IOL: intraocular lens, ACD: anterior chamber depth, AS: anterior segment, ICL: Implantable Collamer Lens.

**Table 2:** Quantitative OCT Metrics and Safety Thresholds in Phakic IOLs.

OCT Parameter	Definition	Clinical Thresholds/Normative Values	Clinical Relevance
Central Vault	Sagittal distance between pIOL and crystalline lens	Ideal: ~500 μm; Normal: 250–750 μm; High: >1000 μm; Low: <250 μm	Key predictor of cataract (low vault) or glaucoma (high vault)
Angle Opening Distance (AOD)/Anterior Chamber Angle (ACA)	Angle width in degrees or AOD500 (μm from scleral spur)	Desirable: ≥30°; Concerning: <20°; Shaffer grade III+ for ICL eligibility	Monitors angle closure risk; guides ICL eligibility and follow-up
Anterior Chamber Depth (ACD)	Distance from endothelium to lens surface (or epithelium to lens)	Pre-op ICL: ≥2.8 mm (endo-lens); Post-op: avoid <2.0 mm	Monitors postoperative shallowing; linked to vault and safety
Cornea–pIOL Distance	Distance from pIOL (center or edge) to corneal endothelium	Central > 2.0 mm; Peripheral > 1.5 mm	Used in iris-claw pIOLs (e.g., Artisan/Artiflex) for endothelial safety monitoring
Crystalline Lens Rise (CLR)	Vertical rise of the lens above angle-to-angle line on horizontal OCT	Higher CLR predicts low vault; Normative CLR ~0–100 μm	Used for ICL sizing and vault prediction
Sulcus-to-Sulcus (STS)/Angle-to-Angle (ATA)	STS via UBM; ATA via OCT as surrogate	STS ~11.5–12.5 mm; ICL–ATA difference (compression): 0.25–0.50 mm	Predicts vault via oversizing; used to estimate ideal lens size
IOL Tilt	Angular deviation of IOL from ocular axis (°)	Normal tilt: <3–5°; Symptomatic if >5–7°	Associated with optical aberrations; guides repositioning decisions
IOL Decentration	Offset of IOL center from visual axis or corneal vertex (mm)	Normal: ~0.2–0.3 mm; >0.5 mm may induce coma/glare	Associated with dysphotopsia if >0.5 mm; monitored for stability
Repeatability/Reproducibility	Inter-test consistency of OCT measurements	Tilt: ±0.3°; Decentration: ±0.05 mm; ICCs > 0.9 for vault/ACD	Confirms reliability of serial measurements and follow-up
Iridocorneal Angle	Angle formed between iris and cornea (non-AOD methods)	AOD > 30°, alert if <20°	Detects narrow angles or peripheral anterior synechiae
Tilt/Decentration (combined)	Displacement or angular misalignment of ICL from ideal position	Tilt < 5°, Decentration < 0.3 mm	Correlates with optical quality and visual symptoms
Other Metrics	ACV: anterior chamber volume; LT: lens thickness	Small ACV → high vault risk Thicker lens → reduced vault space	Adds risk stratification (crowding); integrates with biometry

**Table 3:** Comparison of OCT findings by intraocular lens type or technique.

Lens Type	Surgical Technique	Key OCT Parameters	Common Findings	Clinical Implications
ICL (Implantable Collamer Lens)	Phakic intraocular lens (pIOL) implanted in the posterior chamber (sulcus), requires precise sizing (nomogram, formulas)	Central vault, anterior chamber depth (ACD), iridocorneal angle, lens rise, tilt, decentration	Low vault (<250 μm) → risk of cataract; high vault (>1000 μm) → glaucoma or angle closure	Requires vault monitoring; consider ICL exchange if cataract or angle closure risk; critical sizing
Artisan/Artiflex (pIOL, anterior chamber)	pIOL enclavated to the iris in the anterior chamber (pre- or retropupillary)	pIOL-corneal distance (central and peripheral), ACD, tilt/decentration	Low peripheral vault → endothelial cell loss; tilt/decentration if asymmetric enclavation	Close follow-up if cornea-pIOL distance < 1.5 mm; risk of endothelial loss; consider explantation
Carlevale lens (Secondary, scleral plugs)	Pseudophakic lens fixed with scleral plugs at ~3 mm from the limbus	Plug position in sclera, tilt, decentration, anterior chamber configuration	Well-inserted plugs; possible reverse pupillary block (RPB)	Confirm plug depth; prevent erosion or exposure; monitor for RPB and pressure changes
Yamane technique (Secondary, sutureless intrascleral fixation)	Pseudophakic lens with flanged haptics inserted into scleral tunnels ~2 mm from the limbus	Flange position in scleral tunnel, tilt, decentration, iris configuration	Moderate tilt (~2–4°), mild decentration, flange erosion if poorly positioned	Assess flange stability; detect early malpositions; prevent tilt or dislocation

**Table 4:** OCT-based decision-making in phakic and secondary IOLs. Summary of key anterior segment OCT findings after phakic or secondary intraocular lens implantation, their associated complications, and suggested clinical actions. This table provides a quick reference for clinicians to translate imaging results into practical management strategies.

OCT Finding	Associated Risk	Suggested Clinical Action
Vault < 250 µm (ICL)	Anterior subcapsular cataract (lens–ICL contact)	Close follow-up; consider ICL exchange for larger size
Vault 250–750 µm (ICL)	Safe range	Routine OCT monitoring (6–12 months)
Vault > 1000 µm or narrow angles (ICL)	Angle closure, secondary glaucoma	Monitor IOP and angles; consider PI, lens rotation or downsizing
Cornea–IOL distance < 1.5–2.0 mm (iris-claw pIOLs)	Endothelial cell loss	Intensify endothelial cell counts; consider explantation if progressive loss
Tilt > 5° or Decentration > 0.5 mm	Higher-order aberrations, glare, halos	Repositioning/re-enclavation if symptomatic; observation if stable and asymptomatic
Iris–ICL contact/abnormal iris profile	Pigment dispersion syndrome	Monitor pigment deposits and IOP; consider ICL exchange if progressive or symptomatic
Reverse pupillary block configuration (scleral-fixated IOLs)	IOP elevation, angle shallowing	Perform peripheral iridotomy (prophylactic or therapeutic)
Progressive vault reduction over time	Increased risk of cataract	Annual OCT follow-up; anticipate possible ICL exchange
Peripheral anterior synechiae (pIOLs)	Chronic angle closure, glaucoma	Gonioscopy + OCT monitoring; consider explantation if progressive

## Data Availability

No new data were created or analyzed in this study. Data sharing does not apply to this article.
